# Age-Related Decrements in Heat Dissipation during Physical Activity Occur as Early as the Age of 40

**DOI:** 10.1371/journal.pone.0083148

**Published:** 2013-12-12

**Authors:** Joanie Larose, Pierre Boulay, Ronald J. Sigal, Heather E. Wright, Glen P. Kenny

**Affiliations:** 1 Human and Environmental Physiology Research Unit, School of Human Kinetics, University of Ottawa, Ottawa, Canada; 2 Faculty of Physical Education and Sports, University of Sherbrooke, Sherbrooke, Canada; 3 Clinical Epidemiology Program, Ottawa Hospital Research Institute, Ottawa, Canada; 4 Departments of Medicine, Cardiac Sciences and Community Health Sciences, Faculties of Medicine and Kinesiology, The University of Calgary, Calgary, Canada; Children's National Medical Center, United States of America

## Abstract

Older adults typically experience greater levels of thermal strain during physical efforts in the heat compared to young individuals. While this may be related to an age-dependent reduction in whole-body sweating, no study has clearly delineated at what age this occurs. In the present study, we report direct measurements of human heat dissipation during physical activity in the heat in males ranging in age from 20–70 years. Eighty-five males performed four 15-min bouts of cycling separated by 15-min rest periods, in a calorimeter regulated to 35°C and 20% relative humidity. Direct calorimetry was used to measure total heat loss (whole-body evaporative heat loss and dry heat exchange). We also used indirect calorimetry as a continuous measure of metabolic heat production. Body heat storage was calculated as the temporal summation of heat production and total heat loss over the experimental session. Whole-body sweat rate (WBSR) was calculated from measurements of evaporative heat loss. Males were divided into five age categories for the analysis of WBSR and body heat storage: 20–31 years (n = 18), 40–44 years (n = 15), 45–49 years (n = 15), 50–55 years (n = 21) and 56–70 years (n = 16). Relative to young males, WBSR was reduced in males aged 56–70 during each exercise (all *P*<0.05), in males aged 50–55 during the second (*P* = 0.031) and third exercises (*P* = 0.028) and in males aged 45–49 during the final exercise bout (*P* = 0.046). Although not significantly different, 40–44 years old males also had a lower rate of heat loss compared to younger males. Over the sum of two hours, the change in body heat content was greater in males 40–70 years compared to young males (all *P*<0.05). Our findings suggest that middle-aged and older adults have impairments in heat dissipation when doing physical activity in the heat, thus possibly increasing their risk of heat-related illness under such conditions.

## Introduction

It is generally understood that older adults (≥60 years) experience greater thermal and/or cardiovascular strain during exposure to hot environments [1]–[4], and that this may be heightened when physical activity is performed in the heat [5]. There is evidence to suggest that middle-aged adults also have a reduced capacity to thermoregulate during exercise in the heat relative to younger individuals [6], [7]. While the greater level of thermal strain experienced by older adults may be related to an age-dependent reduction in the body's physiological capacity to dissipate heat [6]–[9], no study to date has clearly delineated at what age this begins to occur.

Engaging in exercise or physical activity is strongly advocated to reduce the risk of many adverse health outcomes that have a tendency to occur as people age [10]. For older individuals, physical activity is often associated with leisure and/or tasks of daily life, many of which are performed outdoors (e.g. gardening, hiking, cycling, mowing the lawn, etc.). In addition, older adults working in physically demanding jobs are often required to carry out physical work under arduous environments, thereby increasing thermal stress. The potential health risks associated with physical activity/work in the heat for the aging population and workforce are exacerbated by extreme heat events which are anticipated to increase in frequency, duration and intensity over the next few decades [5], [11]. In view of the fact that heat related illness (e.g. heat cramps, heat exhaustion, heat stroke) can largely be prevented by identifying individuals who are at risk, the present study examined whole-body heat dissipation and heat storage during intermittent moderate intensity physical activity in the heat in a large sample of healthy males ranging in age from 20 to 70 years. It was hypothesised that aging would be associated with a progressive decline in heat loss capacity, resulting in a greater change in body heat content among older males relative to young males.

## Methods

### Ethics Statement

The experimental procedures were approved by the University of Ottawa Health Sciences and Science Research Ethics Board. Written informed consent was obtained from all volunteers prior to their participation in the study.

### Study population

Eighty-five males volunteered to participate in this study. All participants were healthy and physically active with no history of cardiovascular, metabolic or respiratory disease. Our participants were also not taking medication related to these conditions. Measurements of height, body mass, body density and peak oxygen uptake (VO_2peak_) were determined prior to the experimental session. Measurements of body density were obtained using the hydrostatic weighing technique and were used to calculate body fat percentage [12]. Body surface area was calculated from the measurements of weight and height according to DuBois and DuBois [13]. A progressive cycle ergometer protocol was utilized to determine VO_2peak_. Continuous electrocardiographic monitoring was used during the maximal exercise test in males 50 years of age or above.

### Experimental session

The day prior to the experimental session, participants were instructed to avoid caffeine, alcohol and strenuous exercise, as well as to drink plenty of water. On the day of the experimental session, participants were asked to come to the laboratory after eating a light breakfast and to avoid any major thermal stimuli on their way to the session. Participants were instrumented in a thermoneutral room and then entered the whole-body calorimeter regulated to 35°C and ∼20% relative humidity, where they rested in an upright seated position for a 30-min habituation period while steady-state measurements were obtained. Thereafter, participants performed four 15-min bouts of cycling at a rate of metabolic heat production equal to 400 W (∼80 W external resistance) separated by 15-min rest periods, for a total duration of two hours.

### Measurements of whole-body heat loss and heat storage

We used the world's only direct calorimeter (a device for making extremely accurate measurements of the heat emitted by the human body) to determine total heat loss from measures of evaporative heat loss and dry heat exchange (radiation, convection and conduction). The rates of evaporative heat loss and dry heat exchange were measured as the difference in humidity and temperature, respectively, between the incoming and outgoing air. The following equations were used to calculate evaporative heat loss (*H_E_*) and dry heat exchange (*H_D_*):
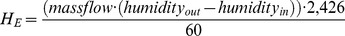


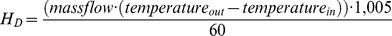
where mass flow is the rate of air mass (kg air·s^−1^), 2,426 is the latent heat of vaporization of sweat (J·g sweat^−1^) and 1,005 is the specific heat of air [J·(kg air·°C)^−1^]. A full peer-reviewed technical description of the performance and calibration characteristics of the whole-body calorimeter is available [14]. We also used indirect calorimetry to estimate the rate of metabolic heat production (*M*) from measurements of oxygen and carbon dioxide concentrations in a known volume of expired gas (Moxus modular metabolic system, AEI Technologies, Bastrop, Texas, USA). We subsequently calculated the change in body heat content as the temporal summation of heat production and total heat loss during each exercise and recovery period, as well as over the two hour experimental session: heat storage = *M*−*H_E_*±*H_D_*. Evaporative heat loss was used to measure whole-body sweat rate in g·min^−1^ which was calculated as: evaporative heat loss (in W) multiplied by 60 s and divided by the latent heat of vaporization of sweat (2,426 J · g of sweat^−1^). Rectal temperature was measured by inserting a thermocouple probe (Mon-a-therm General Purpose Temperature Probe, Mallinckrodt Medical, St. Louis, MO, USA) to a minimum of 12 cm past the anal sphincter.

### Data analysis

In order to obtain a significantly different age gap between young and middle-aged adults, only young males between the ages of 20–31 were recruited (n = 18). Given that previous work showed no differences in whole-body heat loss and heat storage between young and middle-aged males (40–45 years) [16], albeit at a much lower rate of heat production, we assumed that thermoregulatory responses would be similar for males between the ages of 20–39. Conversely, age-related deteriorations in a number of physiological systems (e.g., musculoskeletal, circulatory, endocrine, etc.) are known to occur progressively throughout middle to late adulthood (i.e., 40–70 years), and a number of studies suggest that heat loss capacity also deteriorates during this age span [1]–[9]. To determine when this begins to occur, middle-aged and older adults were divided into four age categories (40–44 years [n = 15], 45–49 years [n = 15], 50–55 years [n = 21] and 56–70 years [n = 16]) for the analysis of whole-body sweat rate and change in body heat content.

### Statistical analysis

The values used for the analyses of whole-body sweat rate, body heat content and rectal temperature were obtained by averaging the last minute of each exercise (Ex1, Ex2, Ex3 and Ex4) and recovery bout (R1, R2, R3 and R4). A mixed-design analysis of variance was used to test for differences in whole-body sweat rate, body heat content and rectal temperature between age groups (five levels) and across the exercise or recovery bouts (four levels). Measurements obtained during the exercise and recovery periods were analysed separately. We also compared the net change in body heat content at the end of the two hour experimental session as well as participant characteristics using one-way analysis of variance. In a secondary analysis, the data for whole-body sweat rate (measured in the final minute of the last exercise period) as well as change in body heat content were pooled (separately) to examine whether these two variables correlated with relevant participant characteristics (i.e. age, VO_2peak_, body surface area and body fat percentage) using Pearson product-moment correlation coefficients. An alpha level ≤0.05 was considered statistically significant. The statistical software package SPSS 20 (SPSS Inc., Chicago, IL, USA) was used for all analyses.

## Results

Participant characteristics are presented in Table 1. Body surface area, body mass, lean body mass and VO_2peak_ (expressed as a function of lean body mass) were not different between age groups. Body fat percentage was significantly greater in males in the age categories 45–49 (*P* = 0.028) and 56–70 years (*P* = 0.001) compared to young males. The values for whole-body sweat rate, body heat content and rectal temperature during the exercise and recovery periods are provided in Table 2.

**Table 1:** Participant characteristics.

Group	n	Age, *years*	A_D_, *m^2^*	Weight, *kg*	BF, *%*	LBM, *kg*	VO_2peak_, *mLO_2_·kgLBM^−1^·min*
20–31	18	25.9±0.7	1.99 (1.67–2.31)	80.6 (59.8–95.5)	17.5 (8.6–30.9)	66.4 (46.9–80.9)	53.1 (41.2–64.7)
40–44	15	41.8±0.5*	2.10 (1.82–2.55)	90.1 (70.1–130.1)	22.8 (12.5–31.1)	68.8 (57.8–89.7)	53.1 (40.3–65.7)
45–49	15	47.1±0.3*§	2.10 (1.88–2.38)	89.3 (73.9–116.0)	24.9 (14.9–37.5)*	66.6 (53.2–78.6)	55.4 (38.3–68.9)
50–55	21	52.2±0.4*§‡	2.05 (1.73–2.29)	87.6 (65.5–101.9)	22.6 (6.9–35.4)	67.4 (52.3–84.8)	49.8 (39.4–62.0)
56–70	16	61.4±1.0*§‡†	2.02 (1.75–2.41)	86.9 (70.8–130.1)	27.0 (12.6–39.8)*	62.7 (52.6–74.7)	47.9 (34.1–57.8)

± SE. Values for all other variables are mean with minimum and maximum for each group. A_D_  =  body surface area; BF  =  body fat; LBM  =  lean body mass; VO_2peak_  =  peak oxygen uptake. * Significantly different than males 20–31 years. § Significantly different than males 40–44 years. ‡ Significantly different than males 45–49 years. † Significantly different than males 50–55 years. Values for age are mean

**Table 2:** Whole-body sweating, body heat content and core temperature during exercise and recovery.

		20–31	40–44	45–49	50–55	56–70
WBSR, *g·min^−1^*	Ex1	8.2±0.2*	7.6±0.3	7.6±0.2	7.4±0.2	7.2±0.2
	R1	3.7±0.2	3.8±0.2	3.8±0.2	3.7±0.1	4.1±0.2
	Ex2	9.2±0.2*§	8.8±0.2	8.5±0.2	8.4±0.2	8.2±0.2
	R2	3.9±0.1	4.2±0.3	4.1±0.2	4.2±0.2	4.3±0.2
	Ex3	9.5±0.2*§	9.1±0.2	8.8±0.2	8.6±0.2	8.7±0.2
	R3	4.1±0.2	4.4±0.3	4.2±0.1	4.3±0.1	4.6±0.2
	Ex4	9.5±0.2*‡	9.1±0.2	8.7±0.1	8.8±0.2	8.6±0.2
	R4	4.0±0.1	4.0±0.3	4.2±0.2	4.3±0.2	4.4±0.2
H_b_, *kJ*	Ex1	146.7±5.6	159.3±6.7	152.6±6.1	162.6±4.2	167.5±5.6
	R1	−38.8±5.9	−21.8±4.4	−26.8±5.3	−38.1±3.4	−36.3±6.4
	Ex2	94.7±4.5*§‡	107.5±6.4	119.2±5.8	120.3±5.1	120.4±5.5
	R2	−67.4±5.2	−50.4±6.7	−56.4±5.5	−63.7±2.8	−66.5±5.0
	Ex3	92.0±4.5*	97.3±4.6	109.9±5.3	107.0±4.1	114.6±4.6
	R3	−72.2±3.2	−58.1±3.3	−61.3±5.1	−69.4±4.4	−71.8±5.2
	Ex4	91.9±3.6*§‡	94.6±5.7	112.1±5.5	110.6±4.0	113.6±4.7
	R4	−86.5±4.3†	−64.2±7.2	−71.1±6.3	−83.6±4.2	−86.0±5.2
T_rec_, °*C*	Ex1	37.27±0.07	37.15±0.10	37.10±0.09	37.19±0.05	37.20±0.08
	R1	37.31±0.08	37.20±0.09	37.18±0.08	37.21±0.05	37.25±0.07
	Ex2	37.48±0.07	37.35±0.09	37.34±0.08	37.38±0.05	37.41±0.07
	R2	37.45±0.08	37.33±0.08	37.36±0.08	37.33±0.05	37.42±0.06
	Ex3	37.59±0.08	37.47±0.08	37.48±0.08	37.47±0.05	37.54±0.06
	R3	37.52±0.07	37.43±0.07	37.46±0.08	37.41±0.05	37.50±0.06
	Ex4	37.65±0.07	37.55±0.08	37.56±0.08	37.54±0.05	37.60±0.05
	R4	37.54±0.08	37.48±0.07	37.55±0.08	37.45±0.05	37.55±0.05

± SE. WBSR  =  whole-body sweat rate; H_b_  =  change in body heat content; T_rec_  =  rectal temperature; Ex  =  exercise; R  =  recovery. * Significant difference between age groups 20–31 and 56–70. § Significant difference between age groups 20–31 and 50–55. ‡ Significant difference between age groups 20–31 and 45–49. † Significant difference between age groups 20–31 and 40–44. Values are mean

### Whole-body sweat rate

**Figure 1:**
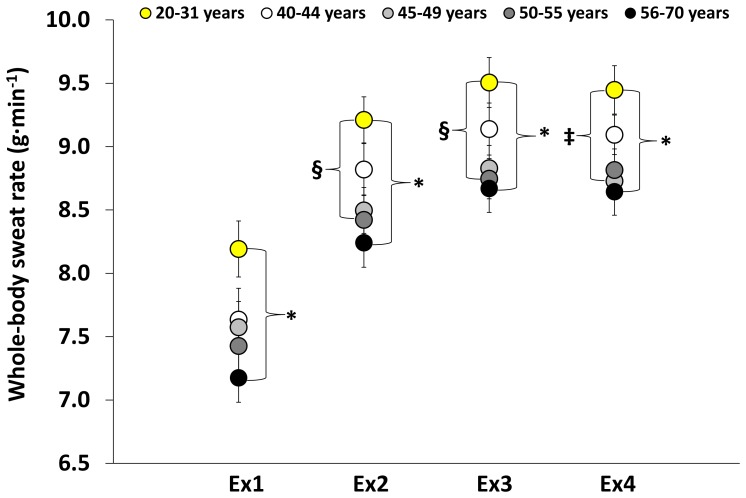
Whole-body sweat rate during repeated bouts of physical activity. Whole-body sweat rates at the end of each exercise bout performed in a direct calorimeter regulated to 35°C and 20% relative humidity. * Significant difference between males 56–70 and 20–31 years. § Significant difference between males 50–55 and 20–31 years. ‡ Significant difference between males 45–49 and 20–31 years. Values are presented as mean ± SE. Significance level accepted at *P*≤0.05.

Whole-body sweat rate was significantly different between groups (*P* = 0.004). In comparison to the youngest age group, whole-body sweat rate was significantly reduced in males aged 56–70 during each exercise (all *P*<0.05), in males 50–55 during the second (*P* = 0.031) and third exercises (*P* = 0.028) and in males 45–49 during the final exercise bout (*P* = 0.046) (Figure 1). Whole-body sweating was also slightly lower in males between 40–44 years of age relative to younger males although this was not statistically significant. During the recovery periods, whole-body sweating was similar between groups.

### Body heat content

As a result of reduced whole-body sweat rate, changes in body heat content also differed between groups during exercise (*P* = 0.001). Heat storage was greater in males 56–70 years of age compared to young males during Ex2 (*P* = 0.012), Ex3 (*P* = 0.008) and Ex4 (*P* = 0.014). Males between the ages of 50–55 and 45–49 stored significantly more heat during Ex2 (*P* = 0.006 and *P* = 0.024 respectively) and Ex4 (*P* = 0.032 and *P* = 0.035 respectively) compared to younger males. There was a significant group difference in the change in body heat content during the recovery periods (*P* = 0.002). Males in the age group 40–44 had a lower negative change in body heat content (i.e., stored more heat) than young males during R4 (*P* = 0.049).

**Figure 2:**
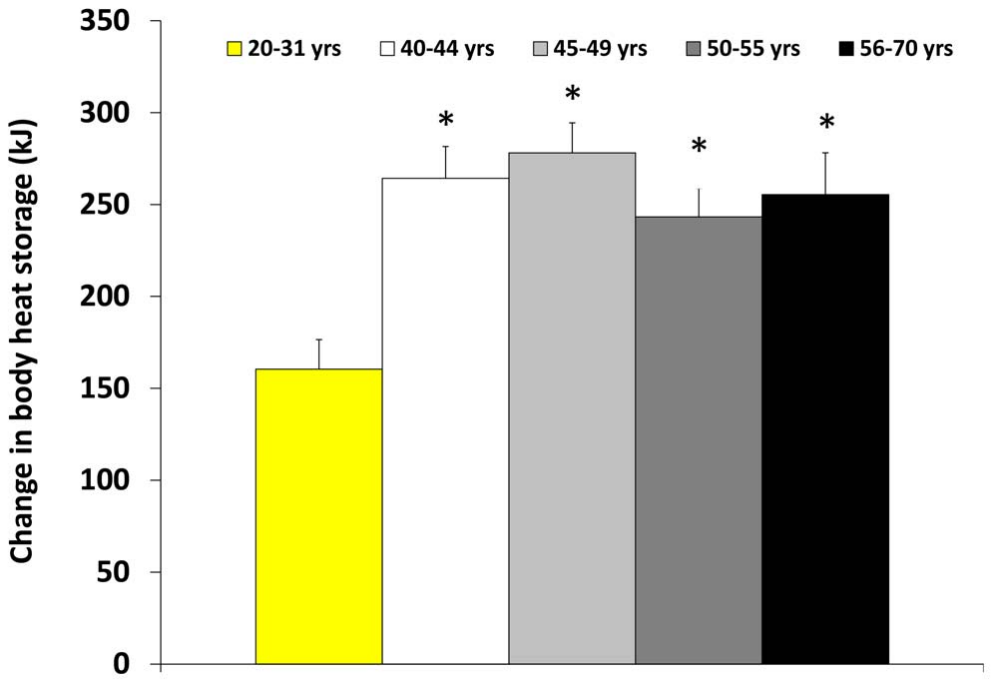
Cumulative change in body heat storage over two hours of physical activity/rest in the heat. Total amount of heat stored in the body after four 15-min bouts of cycling at an external workload of ∼80 W separated by 15-min rest periods at an ambient air temperature of 35°C and 20% relative humidity. * Significantly different from age group 20–31 years. Values are presented as mean ± SE. Significance level accepted at *P*≤0.05.

The cumulative change in body content over the two hour experimental session was significantly greater in males 40–44 (*P* = 0.001), 45–49 (*P*<0.001), 50–55 (*P* = 0.005) and 56–70 years of age (*P* = 0.003) relative to young males (Figure 2).

### Core temperature

Rectal temperature at baseline ranged from 36.98±0.11 to 37.14±0.07°C but was not significantly different among groups. There were no differences in rectal temperature during the exercise or recovery periods between groups. The average increase in core temperature was ∼0.50°C.

### Pearson's correlation

Age was significantly correlated with whole-body sweat rate (*r* = −0.39, *P*<0.001), whereby advancing age was associated with a reduction in whole-body sweating. Age was also correlated with body heat content (*r* = 0.37, *P*<0.001), where advancing age was associated with a greater change in body heat content. VO_2peak_ did not significantly correlate with whole-body sweat rate (*r* = 0.07, *P* = 0.516) or body heat content (*r* = −0.04, *P* = 0.696). Body surface area and body fat percentage both correlated with body heat content (*r* = 0.31, *P* = 0.003; *r* = 0.22, *P* = 0.041, respectively) but not with whole-body sweat rate (*r* = 0.07, *P* = 0.516; *r* = −0.03, *P* = 0.779, respectively).

## Discussion

We found that whole-body sweat rate was significantly reduced in males between the ages of 45–70 years during short bouts (15 minutes) of repeated physical activity in the heat. Interestingly, as depicted in Figure 1, there was also a small disparity in whole-body sweat rate between males in the age group 40–44 and younger males. Noteworthy is the fact that whole-body sweating did not significantly differ between young and older adults during the recovery periods, although middle-aged males (40–49 years) tended to lose less heat at rest than their younger counterparts. The cumulative effect of reduced whole-body heat loss capacity after two hours of alternating between brief periods of physical activity and rest was a greater change in body heat content in males 40–70 compared to young males.

The present study examined thermoregulatory responses across an age continuum and our results suggest that deteriorations in heat loss capacity occur progressively from middle adulthood through to older adulthood. Previous studies have either compared thermoregulatory function during exercise in the heat between young and middle-aged adults [6], [7], [15]–[17] or between young and older adults [8], [9], [18], [19] using relatively small sample sizes. The results and conclusions derived from these studies varied such that some studies reported age-related impairments in sweating capacity in middle-aged [6], [7] and older adults [8], [9], [19], whereas other studies found no differences as a function of age [15]–[18], [20]. It is important to consider that in addition to small sample sizes [18], a number of factors may account for the discrepancies between the results of the present study and those derived from previous work. For example, some studies measured heat loss capacity in highly trained middle-aged and older adults [15]–[17], or failed to ensure the same thermal stimulus between independent age groups (based on differential rates of metabolic heat production) [20]. In addition, some conclusions were based exclusively on measurements of local heat loss responses thereby limiting results to a very small area of the body [19]. The present study findings are in accordance with the studies that demonstrated a greater level of thermal strain in older adults during physical activity in the heat, and that this is likely due to age-related impairments in whole-body sweating capacity. However, our results expand the existing literature by providing evidence that heat loss capacity may be compromised as early as the age of 40–44 and decline progressively thereafter. Previous studies have also reported that factors such as VO_2peak_ and/or body composition had a greater influence on heat loss capacity than “age” *per se* [20], [21]. We demonstrated that whole-body sweating did not correlate with VO_2peak_ (range 34.1 to 68.9 mLO_2_·kg LBM·min^−1^), body surface area (range 1.67 to 2.55 m^2^) or body fat percentage (range 7 to 40%). On the other hand, age was the only variable to significantly correlate with whole-body sweat rate. This would suggest that when the stimulus for sweating is equal for all participants, which we achieved by using a fixed heat load (i.e. 400 W) under fixed environmental conditions, aging may have a larger influence on whole-body heat loss capacity than the fitness level or specific physical characteristics of the individual.

### Strengths and limitations

Our laboratory is equipped with the world's only direct calorimeter, the gold standard for making extremely accurate measurements of the heat emitted by the human body. Therefore the major strength of the present study was that we were able to precisely measure whole-body heat dissipation, and consequently whole-body heat storage, in a large sample of males between the ages of 20 and 70 years. Alternatively, studies in the field of thermoregulatory physiology typically measure local sweat rate as an estimate of whole-body heat dissipation and/or core temperature as an indication of whole-body heat storage. It is important to consider that conclusions based solely on these local measurements could be misleading. For example, our results indicate that the change in rectal temperature after the two hour experimental session was very similar across age groups. Core temperature measurements however, only reflect how much heat is stored within a specific region of the body and have been shown to underestimate how much heat is stored throughout the entire body [22]–[24]. The amount of heat stored over the whole-body after two hours of physical activity/rest in the heat, as measured by direct calorimetry, was actually 35–42% greater in middle-aged and older males compared to young males.

The intensity of the physical activity performed by our participants was very moderate and was done in a dry environment inside a laboratory. Thus, it is unclear if impairments in heat dissipation as a function of age would be more pronounced at higher workloads or on the other hand, if differences would occur at even lower workloads. Our participants were habitually active by nature of their occupation (e.g., firefighters, police officers, military personnel, construction workers), but none reported being highly trained in endurance sports. Nevertheless, it is possible that some of our middle-aged and older participants were more fit than the average population, and that age-related decrements in whole-body heat loss capacity may be greater in sedentary individuals. Finally, it remains to be determined whether thermal strain would be exacerbated for middle-aged and older adults under conditions which restrict evaporative cooling, for example in more humid environmental conditions or with protective clothing.

### Practical Perspective

Older adults are encouraged to engage in regular physical activity as this has been shown to elicit important health benefits that contribute to healthy aging. However, with the predicted increase in the Earth's average temperature combined with the rapidly aging global population and workforce in coming years, a greater proportion of older individuals will be required to work and play in the heat. While adults over the age of 60 are generally regarded as being especially vulnerable in the heat, we showed that people as young as 40 years of age stored a greater amount of heat as a result of reduced heat dissipation in comparison to younger adults. Since middle-aged and older adults appeared to experience similar levels of thermal strain during physical activity in the heat, the present findings might alter the age at which it is recommended that adults take precautionary measures on hot days (e.g. refrain from outdoor activity, increase fluid intake and/or find cooler shelter).

## Conclusion

Decrements in whole-body heat loss capacity were apparent as early as the age of 40 and declined with advancing age. We conclude that not only should older adults be cautious of the risks associated with performing physical activity when ambient air temperature rises, but middle-aged adults should also be aware that they could be more prone to heat-related illness compared to young individuals.
